# Satisfaction with Maternal Healthcare Services in the Ketu South Municipality, Ghana: A Qualitative Case Study

**DOI:** 10.1155/2019/2516469

**Published:** 2019-04-10

**Authors:** Hubert Amu, Samuel H. Nyarko

**Affiliations:** ^1^Department of Population and Behavioural Sciences, School of Public Health, University of Health and Allied Sciences (UHAS), Hohoe, Ghana; ^2^Institute for Demographic and Socioeconomic Research, University of Texas at San Antonio, TX, USA

## Abstract

**Background:**

Women's satisfaction with maternal healthcare services is vital in quality healthcare delivery. However, the dearth of in-depth information on the issue is a challenge in Ghana. In this study, we explore women's satisfaction with maternal care services at a health facility in the Ketu South Municipality, Ghana.

**Methods:**

This is a qualitative study that used a purposive sampling technique to select 15 women who attended a child welfare clinic at the facility for in-depth interviews. The interviews were tape-recorded, and the results presented in quotes in accordance with the themes that emerged.

**Results:**

The study found that respondents were generally satisfied with the quality of maternal healthcare services provided to them. However, they were dissatisfied with drug administration procedures at the facility. Respondents generally reported poor attitudes on the part of healthcare providers at the health facility. Some logistics were also reported to be in unfavorable condition. Nonetheless, respondents generally had positive perceptions about maternal care services provided to them by the healthcare facility.

**Conclusions:**

Drug administration procedures and attitude of healthcare providers toward clients as well as logistics need to be improved to enhance satisfaction with services at the health facility, particularly among pregnant women and mothers.

## 1. Background

The quality of care is considered as a key element of human rights and the route to equity and dignity of women and children [1]. The provision of quality facility-based maternal healthcare, particularly forty-eight hours after delivery, is an important input in saving maternal lives and preventing disabilities [2]. Thus, understanding the experiences and expectations of women across the continuum of antenatal, perinatal, and postnatal care is important in assessing the quality of maternal healthcare and determining problematic areas that require improvement [3]. Patient satisfaction is a dynamic and subjective perception of the extent to which expected healthcare is provided [3]. It reflects the patient's judgment of various aspects of maternal healthcare, including organisational and interpersonal aspects [3].

Globally, attempts to assess and improve the quality of maternal healthcare over the last two decades have led to increasing importance being given to the expectations, opinions, and experiences of users of maternal healthcare [3]. According to Bergstrom, good quality maternal healthcare should include the decision of women who utilise maternal healthcare services and consider them as partners in maternal healthcare provision [4]. Etsey [5] indicates that maternal healthcare must be equipped with essential supplies including drugs and equipment and staffed with healthcare providers that are not judgmental but are respectful and responsive to the needs of women. Bergstrom further contends that it is important to recognise quality maternity care not as a luxury but rather as a way of making services cost-efficient by meeting the needs of women in appropriate ways [4].

Across the maternal healthcare continuum, the assessment of maternal satisfaction has basically focused on the physical environment, availability of services, hygiene and accommodation conditions, interpersonal relationships with healthcare professionals, the organisation of work, and the expertise and competence of healthcare professionals [6]. Satisfaction with maternal healthcare has been noted by Georgsson Ohman et al. as an experience that results from a subjective evaluation of what women expect to happen in terms of maternal healthcare and what actually happened upon utilisation of the services [7].

In Africa, the provider of maternal healthcare has been identified as a key element in client satisfaction [5]. It has been observed that when healthcare providers fail to perform in ways that conform to the expectations of clients, this impacts negatively on client satisfaction [5]. For instance, in Kenya, a study revealed that about 65% women expressed dissatisfaction with maternal healthcare delivery due to the negative attitude of the healthcare provider towards them and long waiting time even with emergency cases [8]. In Ghana, a few studies have attempted to address issues related to satisfaction with maternal healthcare services with most of them focusing on the preference of delivery methods and service providers. Nketiah-Amponsah, for instance, examined the determinants of consumer satisfaction with healthcare services with an emphasis on the choice of healthcare provider [9]. Dzomeku also investigated maternal satisfaction with healthcare services during labour, which also focused on healthcare providers [10]. Much attention has, however, not been given to the experiences of clients regarding their satisfaction with maternal healthcare services provided to them. Meanwhile, according to the WHO, the proportion of all women giving birth in a health facility who express satisfaction with healthcare services is an important standard for improving maternal and child healthcare [11]. It against this backdrop that we seek to explore maternal satisfaction with the prenatal, delivery, and lying-in period at a health facility in the Ketu South Municipality, Ghana.

## 2. Materials and Methods

### 2.1. Study Setting and Design

The study was conducted at a public health facility in the Ketu South Municipality of the Volta Region, Ghana. This health facility provides healthcare services for over 200,000 people within its catchment area. This paper used a qualitative case study design to explore and understand women's satisfaction and personal experiences with maternal healthcare services at the only major public health facility in the municipality.

### 2.2. Study Population

The target population was women who attended a child welfare clinic (CWC) and had received both antenatal and skilled delivery healthcare at the facility with their children they were attending the CWC with. Mothers who attended the child welfare clinic at the healthcare facility but did not attend the antenatal clinic and gave birth at the facility were, however, excluded from the study.

### 2.3. Sampling Procedure

We used a purposive sampling technique to select the respondents for the study. An exit strategy was adopted in purposively selecting the participants. With this, the data collectors waited at the entrance of the building in which the women attended the CWC. After the women were attended to by the health workers and were leaving the facility, the data collectors then asked them whether they had attended ANC with the child(ren) they were attending the CWC with, at the facility. Those who responded in the affirmative were then recruited upon agreeing to participate in the study. The respondents were purposively selected because the target population is rich with information and will help to understand the research problem better [12]. The sample size of the study was based on the principle of thematic saturation [13], where respondents were continuously selected until no new data or theme is generated. Following from this, thematic saturation was attained after the participation of the fifteenth (15th) respondent. Saturation was determined by the interviewers, noting the key issues that were emerging from the interviews being conducted and the process was halted when no new issues were mentioned by the subsequent respondents. The interviewers had the chance to compare notes because the respondents came to the CWC at various times in the day, and this allowed for comparisons of notes to be made after one or two interviews were conducted. In effect, only women who were present at the health facility as of the time of the study were contacted for the study.

### 2.4. Data Collection Process

Data were collected through in-depth interviews with respondents using an interview guide. This was done by two experienced data collectors who were trained to acquaint them with the research instrument and purpose of the study. The interview guide was divided into five sections: A to E. Section A focused on the sociodemographic characteristics of respondents. Section B dealt with satisfaction with maternal care services. Sections C and D centred on satisfaction with the attitude of healthcare providers and availability of maternal healthcare service logistics, respectively, while section E dealt with the perception of respondents toward maternal healthcare services. The interviews were conducted at convenient and secluded places within the health facility determined by the respondents. The duration of each of the interviews conducted ranged between 30 and 45 minutes. The interviews were conducted mainly in the local language (Ewe) and each was fully tape-recorded. Notes were also taken of key points which were emerging from the interviews. Institutional approval was obtained from the health facility while ethical approval was sought from the University of Cape Coast Ethical Review Board before data were collected. Informed consent was sought from respondents before including them in the study. Respondents were also assured of confidentiality and anonymity of information.

### 2.5. Analytical Strategy

After the interview process, the tape recordings were translated from the local language and transcribed in English by native speakers of the local language. The transcripts were then given to English language experts to review the contents to ensure data validity. Texts from the transcripts were then printed and edited. The data analysis was done manually through the thematic analytical approach where emergent themes were generated from the transcripts. The thematic analysis was done by initially assigning preliminary codes to the data in order to describe the content. Patterns or themes in the codes which were mainly determined a priori across the different interviews were then developed. The themes that emerged were then named and reviewed. These themes were exclusively based on the available services in the health facility at the time of the study. Even though the codes were created by only one of the researchers, a peer debriefing process was involved in the coding development process. This helped to reduce the possibility of researcher bias in the development of the codes. The results are, therefore, presented in the form of direct quotes from the respondents for analysis and discussion.

## 3. Results

### 3.1. Sociodemographic Characteristics of Respondents

Sociodemographic characteristics of respondents surveyed by this study were age, marital status, religion, level of education, trimester in which respondents started antenatal clinic attendance, and the number of antenatal visits they made prior to delivery (Table 1). Respondents were generally within their twenties with 10 out of the 15 respondents interviewed being in their twenties while five were in their early thirties. All respondents were in marital unions and 13 were Christians. With regard to education level, 13 of the respondents had Junior High School (JHS) education. One-third (5) of the respondents started attending the antenatal clinic in the third trimester of pregnancy while six started in the second trimester. Most of the respondents had also attended the antenatal clinic more than four times.

### 3.2. Satisfaction with Maternal Care Services

To assess maternal satisfaction with the services provided, a few issues were considered. These included satisfaction with the head-to-toe examination, health education, services relating to drugs, and delivery services. With regard to the head-to-toe examination, all respondents reported that they were satisfied with the services provided to them. One respondent, for instance, said: “*I was very satisfied since it helped me to know my health status” *(Respondent 13, 26 years). Another respondent also replied: “*Oh Yes! I was satisfied with the head-to-toe examination*” (Respondent 15, 33 years). All respondents indicated that they were satisfied with the health education provided. Some even indicated the particular health education topics they were very satisfied with. A respondent, for instance, said:

The HIV/AIDS counseling was quite satisfactory. I am a hairdresser and they taught me how to handle people very well so that they do not give me the virus. So, I think it was very good and I am very satisfied with it. (Respondent 5, 32 years)

Regarding services relating to drugs, all the respondents expressed dissatisfaction. They reported that the waiting time at the pharmacy was too long. Two respondents, for instance, had these to say concerning drug administration:

I am not satisfied with the laboratory and pharmacy services. When we go for drugs and other drug-related services, we join very long queues, wasting too much time over there. (Respondent 8, 26 years)

There is so much time wasting at the pharmacy and sometimes some prescribed drugs are not on the National Health Insurance Scheme and we need to buy the drugs ourselves depending on the condition. (Respondent 2, 25 years)

For delivery services, some respondents reported being satisfied whereas some reported being dissatisfied. A 33-year old respondent reported:

I was not satisfied when I was admitted 2 days before my delivery due to some complications I had. They (health workers) shouted at me whenever I did not do things right. I was not happy at all with the way the delivery service was provided to me. (Respondent 4, 33 years)

### 3.3. Satisfaction with the Attitude of Healthcare Providers

Regarding satisfaction with the attitude of healthcare providers, the respondents were asked to indicate the attitude exhibited towards them by the health professionals during their antenatal, delivery, and postnatal periods at the hospital. They were also asked to indicate the extent of privacy the healthcare providers accorded them during delivery.

With regard to privacy, the respondents generally indicated that they were given the privacy they needed during delivery and were, thus, satisfied with the level of privacy given to them during delivery. Respondent 9, 24 years, for instance, reported: “*Yes! I was provided with privacy.*” Respondent 3 also replied: “*I was provided with privacy. A green sheet was used to cover the place and no person was allowed to enter*” (24 years).

When asked to generally describe the attitude of healthcare providers towards them, most of the respondents reported that they were not satisfied with the attitude of healthcare providers towards them, with only a few reporting satisfaction with the attitude of the healthcare providers towards them. One of the few respondents who expressed satisfaction with the attitude of healthcare providers, for instance, reported:

Generally, for me, I am satisfied with healthcare providers' attitude even though others think nurses shout or do not behave well toward them. (Respondent 3, 24 years)

On the contrary, two of the respondents who were dissatisfied with the attitude of healthcare professionals had these to say:

When I asked the nurse whether she would examine my vagina to check for “pet or wet” [candidiasis or vagina infection], she shouted at me saying “this place is not Benin.” (Respondent 7, 26 years)

I had a problem with my midwife when I came to the maternity ward. I was in labour and was not well, so I forgot my delivery and baby items. The nurse was then angry and shouted at me and finally left me on the delivery bed until the baby came out. I then pleaded with her before she attended to me. (Respondent 10, 27 years)

### 3.4. Satisfaction with Logistics for Maternal Healthcare Services

Additionally, respondents were asked to explain whether they were satisfied with the logistics and the general environment of the labour ward. They were also asked to indicate how pain management was conducted and their satisfaction with the management. Most of the respondents reported that they were satisfied with the logistics and the general environment of the theatre in which they gave birth. One respondent, for instance, said: “*I was satisfied with all procedures and equipment used since they are the ones trained to use the equipment and perform the procedures*” (Respondent 11, 25 years).

One respondent was, however, not satisfied with the logistics used and the general environment of the ward. She complained about the bed on which she gave birth and the mosquito net she used. She explained:

I was not comfortable with the condition of the bed there, especially the mattress I slept on. The mosquito nets also need to be changed because they were not good enough for people to sleep inside them. (Respondent 2, 25 years)

All respondents reported that pains which they experienced after giving birth were generally managed using infusions and reassurances. Respondent 6, for instance, reported: “*The midwife set up an infusion and told me it will be fine. That was how my pain was relieved” *(31 years). Another respondent also said: “*I was given an infusion and a drug. The midwife also reassured me”* (Respondent 8, 26 years).

One respondent was, however, dissatisfied with how her pain was managed. She responded:

I was not satisfied with the way my pain was managed. During labour, I was in a severe pain, but I was not given any injection until I went to the theatre where I was put to sleep. After the operation, I was not given anything to relieve me of my pain. I had to buy a balm to massage my painful neck. (Respondent 4, 33 years)

### 3.5. Perception of Women about Maternal Healthcare Services

In order to examine the perception of respondentsabout maternal healthcare services provided to them, they were asked to describe the appropriateness of HIV and AIDS guidance and counseling during antenatal healthcare. They were also asked if they would advise their friends or relations to access maternal healthcare services from the hospital.

In relation to the appropriateness of HIV and AIDS guidance and counseling during antenatal healthcare, all respondents reported that the services were very appropriate due to the numerous benefits it brought to them. The following were some of the responses from the respondents: “*The HIV and AIDS counseling is appropriate because one becomes aware of the disease and know what to do if she tested positive”* (Respondent 1, 25 years) and “*The HIV/AIDS counselling is appropriate because it helps you know your status and know more about your life”* (Respondent 12, 32 years).

When asked to suggest what they felt should be improved, most of the respondents indicated that the attitude of service providers should change. Respondent 14 responded: “*The attitude of the nurses. The way they shout at patients especially when you fail to quickly bring out your delivery items when asked, needs to change*” (26 years).

Despite the dissatisfaction with the attitude of healthcare providers, when asked whether they will advise their friends and family members to access services at the hospital, they generally reported in the affirmative. Respondent 8 said: “*I would still advise my relatives to come here because, in general, the services provided are satisfactory*” (26 years). Respondent 6 also said: “*I would advise anyone to come here. This is a municipal hospital and most hospitals refer their cases here*.”

## 4. Discussion

Maternal satisfaction with the quality of maternal healthcare services, as noted by Bazant and Koenig, has been increasingly recognised as an important outcome for the healthcare delivery system [14]. Findings from our study revealed that respondents were generally satisfied with the quality of hair-to-toe examination performed on them by the healthcare professionals. Not only did they indicate being satisfied with the quality of the health education given to them, but respondents also mentioned that they were particularly satisfied with the aspect of health education which relates to HIV and AIDS. This is consistent with a study conducted by Bellows et al. [15] in which it was found that many women were very satisfied with the services provided to them.

The respondents, however, expressed dissatisfaction with services related to drug administration. Their reason was that there was unnecessary delay whenever they went for drugs at the hospital's pharmacy. Most respondents interviewed also expressed satisfaction with the quality of delivery services provided to them with only one of them expressing dissatisfaction. Charging of unannounced fees and long waiting time before being attended to by doctors were also the issues the respondents expressed dissatisfaction with. This is consistent with results of a study conducted by Uzochukwu et al. [16] in which they found the determinants of perceived quality of maternal healthcare services as including time spent at a facility and availability of maternal healthcare services.

Satisfaction of women with maternal healthcare has been found to relate to healthcare provider attitude [15]. This paper also finds that respondents are generally dissatisfied with the attitude of healthcare providers notwithstanding their acknowledgment of adequate privacy being given to them particularly during delivery. This echoes the findings of a study on perceived effects of midwives' attitude toward women in labour in Bayelsa State, Nigeria [17]. It, therefore, concludes that midwives were usually not with pregnant women throughout labour and the attitude of the midwives was generally poor, with only 25 percent of women surveyed rating their attitude as being positive [17].

This study further revealed that the majority of the women were satisfied with the logistics and the general environment of the theatres in which they gave birth, albeit a few respondents were dissatisfied. Most of the respondents were also satisfied with the pain management methods used. Furthermore, the women were generally satisfied with the availability of logistics for the provision of maternal healthcare services in the health facility. This is in direct support of some extant studies in which logistics were found to be available and clients were generally satisfied with the physical infrastructure and diagnostic equipment for ANC and delivery services [18].

Additionally, findings from this study reveal that respondents regarded HIV and AIDS guidance and counseling during their antenatal healthcare as appropriate. They also reported that they would advise their friends and family members to utilise maternal healthcare services provided by the hospital. This, therefore, implies that the women generally had positive perceptions towards maternal healthcare services provided to them. This is consistent with the findings of Fotso and Mukiira [19]. Their findings show a pattern of women's good perception in terms of access to and quality of healthcare services provided by privately owned, substandard, and often unlicensed clinics as well as maternity homes located within their communities [19]. It has also been observed that more than half of women would recommend to friends and family members as well as deliver again in the same facility when they are satisfied with the services rendered to them [15]. However, in the context of this study, it is noteworthy that women may recommend the maternal healthcare services of this health facility not for the sake of satisfaction per se, but for the reason that it is the only referral health facility they have in the municipality.

Despite the important findings made in the present study, the possible weaknesses inherent in the qualitative design adopted are worth mentioning. For instance, the use of purposive sampling technique and the fact that respondents were interviewed at the premises of the facility could have resulted in selection and response biases, respectively. Nevertheless, due to the fact that the respondents willingly agreed to participate in the study before they were recruited and the fact that interviews were conducted at convenient and secluded places within the health facility determined by the respondents, we believe that the possibility of response bias was largely reduced. The use of the exit strategy in recruiting the participants also greatly reduced the level of selection bias.

## 5. Conclusions

Women who accessed maternal healthcare services at the healthcare facility were generally satisfied with the quality of services provided to them, in terms of hair-to-toe examinations, health education, and delivery services. Most of the women were, however, dissatisfied with drug administration procedures. The attitude of healthcare providers toward women who accessed maternal healthcare services at the health facility was found to be generally unfriendly. Some of the logistics of the health facility including beds were reported to be in unfavorable conditions. In spite of the various challenges at the health facility, the women generally had positive perceptions about maternal healthcare services provided to them and were willing to revisit the healthcare facility and recommend it to others. Based on our findings, we recommend that the Ghana Health Service should improve upon the drug administration procedures in the health facilities in the municipality to reduce delay. The attitude of healthcare providers toward women and the logistics also need to be improved in order to improve upon client satisfaction with maternal healthcare services in the health facility.

## Figures and Tables

**Table 1:** Sociodemographic characteristics of respondents.

Respondent	Age	Marital status	Religion	Education	Trimester started ANC	ANC visits
1	25	Married	Muslim	JHS	3rd trimester	4
2	25	Married	Christian	Primary	3rd trimester	5
3	24	Married	Christian	JHS	3rd trimester	4
4	33	Married	Christian	JHS	2nd trimester	7
5	32	Married	Christian	JHS	2nd trimester	6
6	31	Married	Christian	SHS	2nd trimester	7
7	26	Married	Christian	JHS	1st trimester	5
8	26	Married	Christian	None	2nd trimester	4
9	24	Married	Christian	JHS	3rd trimester	4
10	27	Married	Christian	JHS	1st trimester	6
11	25	Married	Muslim	JHS	3rd trimester	4
12	32	Married	Christian	JHS	2nd trimester	6
13	26	Married	Christian	None	2nd trimester	4
14	26	Married	Christian	JHS	1st trimester	5
15	33	Married	Christian	JHS	1st trimester	6

Note: ANC = antenatal care, JHS = Junior High School, and SHS = Senior High School.

## Data Availability

The dataset used to support the findings of this study are contained in this manuscript.

## References

[1] Independent Expert Review Group (iERG) Every Woman, Every Child: Strengthening Equity and Dignity through Health: the second report of the independent Expert Review Group (iERG) on Information and Accountability for Women's and Children's health.

[2] Belle-Brown J., Beckhoff C., Bickford J. (2009). Women and their partner's perceptions of the key roles of the labour and delivery nurse. *Clinical Nursing Research*.

[3] Matejić B., Milićević M. T., Vasić V., Djikanović B. (2014). Maternal satisfaction with organized perinatal care in Serbian public hospitals. *BMC Pregnancy and Childbirth*.

[4] Bergstrom S. (2003). *Maternity Care in Developing Countries*.

[5] Etsey P. M. (2008). *Towards the Millennium Development Goal 5: Assessing the Quality of Supervised Delivery in the Ga West District, Ghana*.

[6] Martinez M. G. (2008). History of art in childbirth in family environment. *Cultura Cuidados*.

[7] Georgsson Öhman S., Grunewald C., Waldenström U. (2009). Perception of risk in relation to ultrasound screening for Down's syndrome during pregnancy. *Midwifery*.

[8] Birungi H., Onyango-Ouma W. (2006). *Acceptability and Sustainability of the WHO Focused Antenatal Care Package in Kenya*.

[9] Nketiah-Amponsah E. (2009). Determinants of consumer satisfaction of health care in Ghana: does choice of health care provider matter?. *Global Journal of Health Science*.

[10] Dzomeku M. V. (2011). Maternal satisfaction with care during labour: a case study of the Mampong-Ashanti district hospital maternity unit in Ghana. *International Journal of Nursing and Midwifery*.

[11] WHO (2016). *Standards for Improving Quality of Maternal and New Born Care in Health Facilities*.

[12] Gliner J. A., Morgan G. A., Leech N. L. (2017). *Research Methods in Applied Settings: An Integrated Approach to Design and Analysis*.

[13] Charmz K. (2003). *Grounded Theory: Objectivist and Constructivist Methods*.

[14] Bazant E. S., Koenig M. A. (2009). Women's satisfaction with delivery care in Nairobi's informal settlements. *International Journal for Quality in Health Care*.

[15] Bellows B., Kyobutungi C., Mutua M. K., Warren C., Ezeh A. (2013). Increase in facility-based deliveries associated with a maternal health voucher programme in informal settlements in Nairobi, Kenya. *Health Policy and Planning*.

[16] Uzochukwu B. S. C., Onwujekwe O. E., Akpala C. O. (2004). Community satisfaction with the quality of maternal and child health services in Southeast nigeria. *East African Medical Journal*.

[17] Onasoga O. A., Opiah M. M., Osaji T. A., Iwolisi A. (2012). Perceived effects of midwives’ attitude towards women in labour in Bayelsa State, Nigeria. *Applied Science Research*.

[18] Tetui M., Ekirapa E. K., Bua J., Mutebi A., Tweheyo R., Waiswa P. (2012). Quality of antenatal care services in eastern uganda: Implications for interventions. *Pan African Medical Journal*.

[19] Fotso J. C., Mukiira C. (2012). Perceived quality of and access to care among poor urban women in Kenya and their utilization of delivery care: harnessing the potential of private clinics?. *Health Policy and Planning*.

